# Evaporative flux method of leaf hydraulic conductance estimation: sources of uncertainty and reporting format recommendation

**DOI:** 10.1186/s13007-022-00888-w

**Published:** 2022-05-12

**Authors:** Xiaoxiao Wang, Jinfang Zhao, Jianliang Huang, Shaobing Peng, Dongliang Xiong

**Affiliations:** National Key Laboratory of Crop Genetic Improvement, MOA Key Laboratory of Crop Ecophysiology and Farming System in the Middle Reaches of the Yangtze River, College of Plant Science and Technology, Huazhong Agricultural University, Wuhan, 430070 Hubei China

**Keywords:** Leaf hydraulic conductance, Evaporation flux method, Rehydration, Gravity pressure, Degassed water, Steady state, Report format

## Abstract

**Background:**

The accurate estimation of leaf hydraulic conductance (*K*_leaf_) is important for revealing leaf physiological characteristics and function. However, the *K*_leaf_ values are largely incomparable in previous studies for a given species indicating some uncertain influencing factors in *K*_leaf_ measurement.

**Result:**

We investigated the potential impacts of plant sampling method, measurement setup, environmental factors, and transpiration steady state identification on *K*_leaf_ estimation in *Oryza sativa* and *Cinnamomum camphora* using evaporation flux method (EFM). The effects of sampling and rehydration time, the small gravity pressure gradients between water sources and leaves, and water degassing on *K*_leaf_ estimation were negligible. As expected, the estimated steady flow rate (*E*) was significantly affected by multiple environmental factors including airflow around leaf, photosynthetically active radiation (PARa) on leaf surfaces and air temperature. *K*_leaf_ decreased by 40% when PARa declined from 1000 to 500 µmol m^−2^ s^−1^ and decreased by 15.1% when air temperature increased from 27 to 37 °C. In addition, accurate steady-state flow rate identification and leaf water potential measurement were important for *K*_leaf_ estimation.

**Conclusions:**

Based on the analysis of influencing factors, we provided a format for reporting the metadata of EFM-based *K*_leaf_ to achieve greater comparability among studies and interpretation of differences.

**Supplementary Information:**

The online version contains supplementary material available at 10.1186/s13007-022-00888-w.

## Highlights

The influences of measurement setup, environmental factors, and steady state identification on leaf hydraulic conductance measurement were estimated and reporting format of *K*_leaf_ were proposed.

**Supplementary Information:**

The online version contains supplementary material available at 10.1186/s13007-022-00888-w.

## Background

Plant hydraulic properties strongly influence photosynthesis and growth. At a given soil water potential, the capacity of leaves to maintain stomata open for photosynthesis mainly depends on plant hydraulic conductance [1]. On average, the hydraulic resistance in leaves accounts for 30% of that in whole-plant, thus leaves constitute an important bottleneck for hydraulic conductance [2]. Due to the important role of leaf hydraulic conductance (*K*_leaf_) in plant, *K*_leaf_ has been extensively studied over the last decades [3–7]. The efficiency of water transport from the petiole to the sites of evaporation through the leaf tissues is quantified as *K*_leaf_, and it is generally expressed as water transport efficiency per unit leaf area (mmol m^−2^ s^−1^ MPa^−1^). A number of approaches have been used to estimate *K*_leaf_ based on excised leaves, such as the evaporative flux method (EFM), the rehydration kinetics methods (RKM), the high-pressure flowmeter (HPFM), and the vacuum pump method (VPM) etc.

The *K*_leaf_ is typically estimated by measuring the ratio of water uptake rate through the leaf to the driving force (e.g., water potential gradient between the petiole to evaporation sites, ΔP). Currently, many methods developed to measure *K*_leaf_, and the EFM has advantage of mimicking the natural transpiration pathways of water movement in the leaf [5]. Furthermore, the EFM method allows other functional traits, such as CO_2_ assimilation rate and stomatal conductance, to be measured simultaneously. Actually, the EFM has been used to measure stomatal conductance by recording the air humidity, air temperature around the leaf and steady-state water flow rate [8]. As the *K*_leaf_ has also been used to explain a range of physiological processes related to photosynthesis, drought tolerance and leaf economical spectrum [9–11], simultaneous estimation of *K*_leaf_ and other traits will provide more reliable information for understanding plant performance under variable conditions.

While the EFM was used in estimating *K*_leaf_ frequently, the *K*_leaf_ values estimated by EFM from different groups are largely incomparable even in the same species (Additional file 1: Fig. S1). Great difference in *K*_leaf_ has been found in the model species, *Arabidopsis thaliana*. In some studies [12–14], *K*_leaf_ of the Columbia (a widely selected ecotype of *A. thatliana*) was less than 10 mmol m^−2^ s^−1^, but it was larger than 50 mmol m^−2^ s^−1^ in other studies [15]. Surprisingly, the huge difference in *K*_leaf_ values estimated using EFM was even found in a *Oryza sativa* genotype, Shanyou 63, and the *K*_leaf_ values varied greatly from 0.64 to 23 mmol m^−2^ s^−1^ in previous studies [16–19] (more details in Additional file 1: Fig. S1 and Additional file 2: Table S1).

Different *K*_leaf_ among studies may be induced by multiple growth environmental factors such as light, temperature, humidity during plant growth. However, it seems unlikely that growth conditions in these studies could have led to such huge differences in *K*_leaf_ values [19–21]. Scoffoni et al. estimated the *K*_leaf_ of six species of lobeliads grown in two irradiances (daily average of 300 vs 800 μmol photons m^−2^ s^−1^) and found the largest variation was only 2.5‐fold in *K*_leaf_ [21]. Alternately, the differences in *K*_leaf_ among studies may be partially due to measurement bias. The environmental irradiance and temperature can influence *K*_leaf_ measurement as shown on a range of species, and they must be controlled accurately [16, 22]. However, the more comprehensive sources of uncertainty in using EFM have been less investigated, and, in fact, the details of the *K*_leaf_ measurements such as the sampling time, sample selection criteria, temperature, photosynthetically active radiation (PARa) and solutions used for *K*_leaf_ measurement were not all available in many studies [16, 17, 20, 23–26], raising the need for the establishment of transparent and detailed method descriptions and protocols.

The lack of *K*_leaf_ estimation and reporting format makes the full and efficient use of *K*_leaf_ from other studies difficult. It is essential to explore the estimation and the reporting format of *K*_leaf_ for unifying and normalizing *K*_leaf_ data from different sources. The study aims to investigate the effects of interference factors during measurement on initial(ψ_initial_) and final leaf water potential (ψ_final_), flow rate, transpiration rate and leaf hydraulic conductance (*K*_leaf_) and to provide the detail recommendations for EFM application and results report.

## Results

**Fig. 1:**
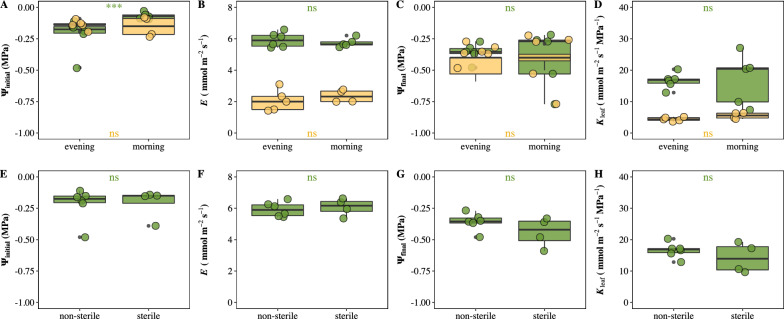
**A**–**D** Influences of sampling time on initial water potential (ψ_initial_), steady flow rate (*E*), leaf hydraulic conductance (*K*_leaf_), and final water potential (ψ_final_), and **E**–**H** the impacts of storage water sterilization on ψ_initial_, *E*, *K*_leaf_, and ψ_final_ of previous sampled leaves. Green points and yellow points represent *O. sativa* and *C.camphora* leaves, respectively. Each point represents one individual leaf. Note, storage water sterilization effects were only investigated in *O. sativa*. (*ns* no significance; ***P < 0.001)

*K*_leaf_ measurements were performed in *O. sativa* and *C. camphora* leaves collected at different daily time to test the impacts of sampling time and the sample storage in the lab. The ψ_initial_ of the samples rehydrated in the lab overnight was significantly lower than the one sampled in the morning of the measurement day in *O. sativa* but not in *C. camphora* (Fig. 1a). However, the decreased ψ_initial_ of the over nightly hydrated samples had no influence on *K*_leaf_. Actually, no differences in both *E* and final leaf water potential (ψ_final_) were observed between the leaves sampled at the previous night and ones sampled in the morning of the measurement day. The water potentials of nightly sampled leaves were measured three times along the rehydration process. The results showed that ψ_initial_ of leaves sampled at previous night was high and convergent within the first 12 h but decreased after 22 h of hydration. Furthermore, recutting in the morning or storage in sterile water alleviated this decrease (Additional file 1: Fig. S3, Fig. 1). No differences in ψ_initial,_ *E*, ψ_final_, and *K*_leaf_ were observed between the *O. sativa* leaves rehydrated in sterile water and the leaves rehydrated in non-sterile water (Fig. 1).

**Fig. 2:**
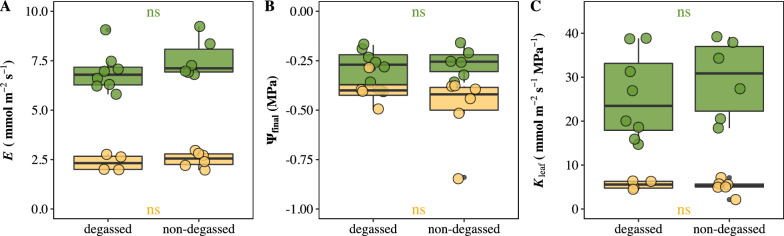
Influences of water degassing on **A** steady flow rate (*E*), **B** leaf hydraulic conductance (*K*_leaf_), and **C** final water potential (ψ_final_). Green points and yellow points represent *O. sativa* and *C. camphora* leaves, respectively. Each point represents one individual leaf. (*ns* no significance)

**Fig. 3:**
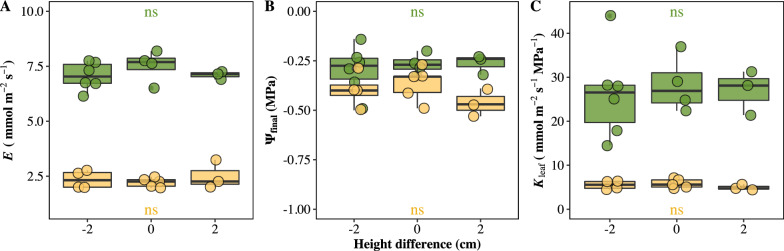
Impacts of height difference between leaf and water surface in cylinder on **A** steady flow rate (*E*), and **B** final water potential (ψ_final_), and **C** leaf hydraulic conductance (*K*_leaf_). 0 indicates same height level between leaf and water surface; − 2 indicates that water surface is 2 cm lower than leaf; 2 indicates that water surface is 2 cm higher than leaf. Green points and yellow points represent *O. sativa* and *C. camphora* leaves, respectively. Each point represents one individual leaf. (*ns* no significance)

We evaluated the impacts of water degassing on *K*_leaf_ estimation by comparing degassed water and distilled water used as water source in the cylinder. Our data showed that *E*, ψ_final_, and *K*_leaf_ estimations in both species were not impacted by water degassing (Fig. 2). Then, the influences of height gradients between water source and leaf blade were investigated, since the height pressure difference may exist between leaf and water source in cylinder. We found that 2 cm height gradient between leaf and water surface in cylinder exhibited no effect on *K*_leaf_ measurement (Fig. 3). In addition, the influences of cylinder water evaporation on *E* estimation under multiple conditions was quantified. The evaporation rate of water in the cylinder without any intervene on water was 0.115 × 10^−3^ mmol s^−1^. The evaporation rate in the cylinder was significantly declined by covering the water surface using liquid wax and/or by maintaining high humidity in weighting chamber (ANOVA, P < 0.001) (Additional file 1: Fig. S2).

The effects of environmental factors including PARa, air temperature, and the airflow through leaf surface on *O. sativa K*_leaf_ estimation were investigated. *K*_leaf_ at 1000 µmol m^−2^ s^−1^ PARa was significantly higher than the values at 500 or 1500 µmol m^−2^ s^−1^. The higher *K*_leaf_ under 1000 µmol m^−2^ s^−1^ PARa was caused by the higher *E* and higher ψ_final_. Low *K*_leaf_ were found under high air temperature condition due to the declined ψ_final_. Interestingly, although the airflow had the strong effects on transpiration, the *K*_leaf_ values estimated under different airflow rates were not significant (Table 1).

**Table 1:** Influences of ambient photosynthetically active radiation (PARa), temperatureand airflow rate on *E*, ψ_final_ and *K*_leaf_ in *O. sativa*

Environment	*E* (mmol m^−2^ s^−1^)		Ψ_final_ (MPa)		*K*_leaf_ (mmol m^−2^ s^−1^ MPa^−1^)	
PARa (µmol m^−2^ s^−1^)						
500	5.68 ± 0.147	***	− 0.32 ± 0.023	ns	16.0 ± 1.13	*
1000	8.75 ± 0.328		− 0.31 ± 0.01		26.8 ± 1.49	
1500	9.22 ± 0.707		− 0.38 ± 0.04		18.7 ± 2.28	
T_air_ (°C)						
37	10.00 ± 0.361	***	− 0.36 ± 0.01	ns	23.0 ± 1.03	*
27	7.01 ± 0.308		− 0.29 ± 0.03		27.1 ± 1.01	
Airflow (m s^−1^)						
1.0	5.14 ± 0.111	*	− 0.28 ± 0.03	ns	20.9 ± 2.91	ns
0	4.66 ± 0.157		− 0.34 ± 0.04		16.6 ± 3.02	

mean ± se, significant differences are indicated: ***P < 0.001; **P < 0.01; *P < 0.05

ns, no significance

**Fig. 4:**
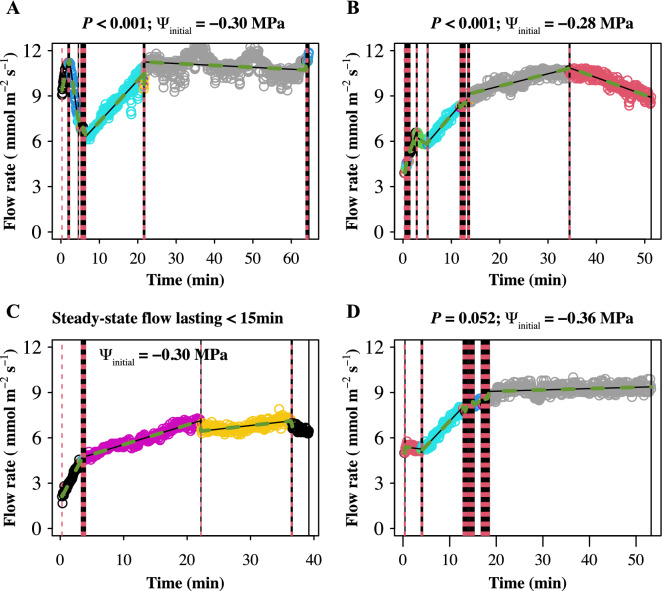
Four typical curves and segment of flow rate to time during measurement. Different segments were marked with different color. **A**, **B** Non-steady curves identified as the slope of last 15 min segment was not equal to zero. **C** Non-steady curve identified as measurement time corresponding to the last segment was less than 15 min. **D** Steady curve, the last segment was longer than 15 min and the slope of last 15 min segment was equal to 0. P represents the P-value of in the last 15 min segment (see details in “Materials and methods” section). ψ_final_ is the initial leaf water potential

**Fig. 5:**
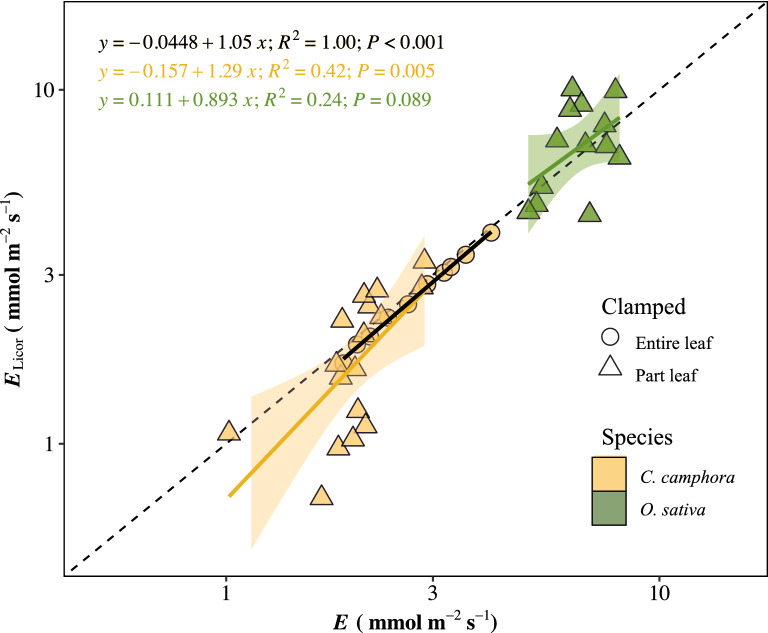
Correlations of stable flow rate measured by balance (*E*) and by gas exchange system Licor 6800 (*E*_licor_) in *O. sativa* (green) and *C. camphora* (yellow). Entire (circle) and partial leaves (triangle) were clamped in gas exchange chamber, respectively. The fitted equations and their p-values for camphor and rice are presented in the upper left of the figure. Blue/red/black fitting line and equation represents the correlation in partly clamped *O. sativa* leaves/partly clamped *C. camphora* leaves/entirely clamped *C. camphora* leaves, respectively

**Fig. 6:**
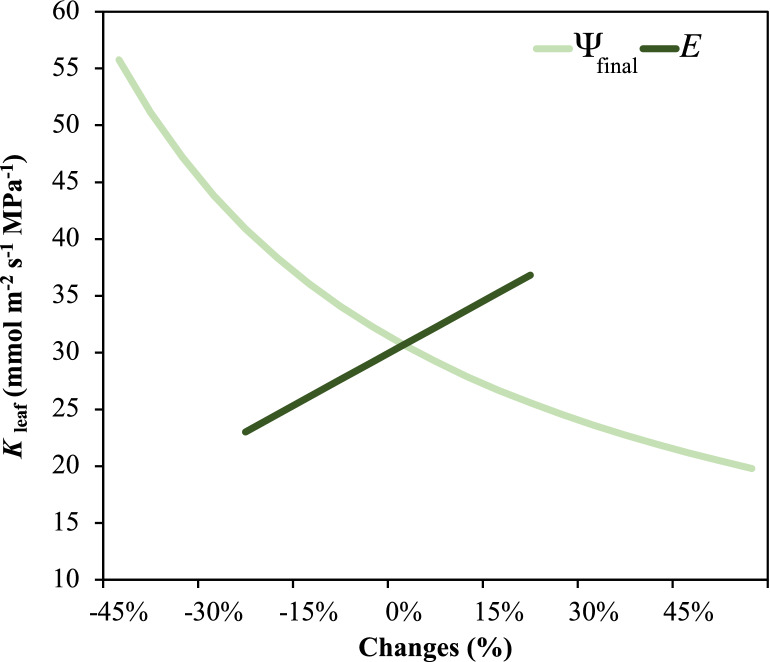
Sensitivity analysis of the influences of final water potential (ψ_final_) and steady flow rate (*E*) on *K*_leaf_. X-axis 0% represents mean value of ψ_final_ and* E* in *O. sativa* measured under 1000 µmol m^−2^ s^−1^ PARa. The x-axis ranges of ψ_final_ and *E* represent their measured value change ranges, respectively

Identifying steady state of flow rate is important because the *K*_leaf_ is directly calculated by using the steady water flow rate through leaves. However, our data indicated that the flow rates of some leaves were oscillated and downward, which did not conform to our steady state criteria (Fig. 4). In the current study, about two of three measurements achieved the continuous and steady state of flow rate according to our criteria (Fig. 4 and supplementary raw data of flow rate). The stabilization of flow rate was further confirmed by the estimation of transpiration rate using the gas exchange system (Additional file 1: Figs. S4, S5, S7), and the flow rate values from the two recording systems were only consistent when the leaves were entirely covered by the gas exchange chamber (Fig. 5). Importantly, the variation of ψ_final_ greatly affected *K*_leaf_. At a given *E* (e.g., using the *E* of 8.7 mmol m^−2^ s^−1^ in *O. sativa* under PARa of 1000 µmol m^−2^ s^−1^), *K*_leaf_ decreased sharply (up to 3-folds) with the decreased ψ_final_, especially within the typical *O. sativa* water potential range of − 0.17 to − 0.45 MPa we observed in the present study, which showed the inverse correlation between K_leaf_ and ψ_final_ (Fig. 6). The ψ_final_ of leaves acclimated at both 0 and 1000 µmol m^−2^ s^−1^ irradiance in advance decreased after 60 and 150 min equilibration in zip-lop bag, respectively (Additional file 1: Fig. S8). Conservatively, we showed that 10 min ~ 1 h equilibration time was proper for water potential measurement.

According to our evaluation, we proposed a reporting format for *K*_leaf_ based on EFM to improve reuse and reanalysis valuable data. Not only the parameters used to calculate *K*_leaf_ but also the raw data of flow rate relative to time should be conserved and provided to meet the needs for identifying flow rate stabilization. Moreover, the environmental factors including airflow, temperature and PARa also should be provided as the *K*_leaf_ estimation was strongly affected by those factors (Additional file 3: Table S2, Additional file 4: Table S3).

## Discussion

In EFM, *K*_leaf_ is calculated by the ratio of water flow rate to the water potential gradient driving water movement across the leaf, and we showed that leaf water potential and *E* were potentially influenced by environmental factors and the criterion for determining physiological stabilization. Our investigation suggested that the remarkable variance in *K*_leaf_ among previous studies might be attributed to methodological artifacts. Therefore, providing details relating to *K*_leaf_ measurement is important to synthesize and to compare data across different studies_._

### Influences of sample collection and storage on *K*_leaf_ estimation

Although the field material sampling at the night before the measurement day and rehydrating overnight in the lab are common practice in previous studies [5, 26–29], to the best of our knowledges, no study investigated the impacts of sampling and storage time on *K*_leaf_ estimation. The tension in xylem is created by transpiration, but the excessive tension in the daytime poses the inherent risk for xylem cavitation, thus substantially reducing the hydraulic conductivity of plants [30, 31]. However, no difference in *K*_leaf_ between leaves sampled at the previous night and leaves sampled in the morning was observed in both species. Rehydration overnight was projected to refill cavitated xylem conduits and restore the lost water in tissues caused by transpiration [32]. Interestingly, our data showed that the ψ_initial_ of overnight rehydrated *O. sativa* leaves was more negative than that leaves sampled in the morning, which wasn’t observed in *C. camphora* (Fig. 1). The more negative ψ_initial_ of leaves rehydrated overnight may result from xylem being blocked by mechanical wound secreta at the cut, such as callose, suberin, lignin, chitinase, and various phenolics [33–35]. Another explanation may be accumulation of sucrose at the cut surface caused by damaged cell wall integrity [36, 37], accelerating microbe attack and water uptake pathway blockage. Indeed, the ψ_initial_ of the overnight rehydrated leaves increased quickly after recutting (Additional file 1: Fig. S3), which might be attributed to the reversible outside-xylem dehydration [38]. Further efforts are still needed to identify the types of microbes and/or the components of the blockage.

### Effects of degassing and gravity pressure on ***K***_leaf_ estimation

Water transport in plants occurs under tension, the air-seeding and its expansion in water will create air-vapor embolisms and thus blocked water transportation. To avoid embolisms, the degassed water is used for *K*_leaf_ measurement in some previous studies [22, 39]. However, no study compared the *K*_leaf_ estimations using degassed water and non-degassed water. Our results showed that degassing had limited influence on *K*_leaf_ estimation, which indicates that gas dissolved in water does not necessarily causing xylem embolism. Unfortunately, no direct evidence has been detected in this study and in previous studies. The EFM follows the natural transpiration-driven water movement pathway in leaves, and the water enters the leaf through internal transpiration driving rather than through external water gravity pressure which may be caused by lower position of leaves than the water meniscus in the cylinder. However, the relative position difference between leaves and water source was generally ignored in previous studies [28, 29]. In this study, the estimated *K*_leaf_ showed no difference when the leaf was placed below, as high as, or above the meniscus of water in the cylinder. The result indicated that the small height difference between leaves and water source was permissible for *K*_leaf_ measurement. Indeed, only 0.196 Pa extra gravity pressure to leaf was changed in the study (Fig. 3).

### Flow rate stabilization identification and water potential measurement

In previous studies, the flow rate stabilization was generally claimed a coefficient of variation < 5% over 3–10 min [22, 28, 40], but no study provided the flow rate variation over time. Apparently, this criterion was not suitable in our *K*_leaf_ measurement. Stricter criteria (P > 0.001, 15 min) for identifying steady state of flow rate were applied in this study. Unexpectedly, we found that the flow rate dramatically oscillated or even reduced after a short-term increase in the measurement of some leaves (Fig. 4). Stomatal oscillations have frequently been observed in previous studies due to the hydraulic mismatch between leaf water transpiration and water supply by xylem [41]. The simultaneous estimation of liquid flow rate and transpiration rate supported this mismatching hypothesis (Additional file 1: Figs. S5, S7), and the oscillation was frequent in high transpiration condition (such as 37 °C condition in Additional file 1: Fig. S6). The declined flow rate after reaching a peak might result from disruption of ionic homeostasis, which might be further attributed to the wide use of deionized water in EFM. Bundle sheath cells permeability have been reported to be related to xylem pH, ionic concentration and component [42–44]. Since the flow rate is highly dynamic over the measurement, and the identification of steady-state flow rate influences *K*_leaf_ estimation, reporting the details of flow rate estimation as well as the raw data of flow rate will be helpful for researchers to interpret the results and syntheses data for meta-analysis in the future.

Besides *E*, the *K*_leaf_ was also strongly affected by ψ_final_. In this study, the ψ_final_ was generally higher than − 0.5 MPa, and, for a leaf with such a high leaf potential, a small error in ψ_final_ estimation would result a large change in *K*_leaf_, emphasizing the importance of accuracy ψ_final_ measurement (Fig. 6). Water potential is typically measured using pressure chamber technique, and the methodological artifacts have been discussed for decades [45–49]. For instance, Levin [46] reported that the contrasting results were obtained by different operators. In addition, it is suggested that leaves need to be equilibrated in bags before the water potential measurement. However, the effects of equilibration time have rarely been reported in literature [46]. In this study, the *O. sativa* leaves sampled under dark and light had different ψ_final_, but leaves sampled under both conditions rapidly achieved water potential equilibrium in bags (Additional file 1: Fig. S8). Further works on improving the accuracy of water potential estimation are needed.

Interestingly, the liquid flow rate and transpiration rate were equal when the entire leaves were clamped in the gas exchange chamber (Fig. 5). The shifted correlations of two-phase water flow rates for partly clamped leaves may be caused by the heterogeneity of transpiration along leaf blade [50]. Our result reminds us of the cautious use of in situ* K*_leaf_ measurement with photosynthetic instruments, another widely used method [27, 51].

## Conclusions

We investigated the potential methodological artifacts in *K*_leaf_ estimation using EFM and showed that environmental factors, such as PARa, air temperature and airflow around leaf, identifications of steady-state flow rate, and ψ_final_ significantly affected *K*_leaf_ estimation. It is important to consider the environmental settings, the flow rate stabilization and precise water potential measurement when estimating *K*_leaf_. In parallel, providing the details of the measurements is also necessary with greater expectations for data preservation, reproducible and open research [52, 53]. We recommend a table-like format (Additional file 3: Table S2) convenient for *K*_leaf_ data measurement and storage.

## Methods

### Plant materials

All the plant materials grew outdoors in Huazhong Agricultural University, Wuhan, China (114^o^22′E, 30^o^29′N). A monocot species, *Oryza sativa* L., *cv* Huanghuazhan (HHZ), was selected and the *O. sativa* plants were growing in paddy field for 50–70 days before sampling. *O. sativa* plants were well watered and fertilized, free of diseases, pests, and weeds. Meanwhile, a dicot species, *Cinnamomum camphora* L. was selected on campus of Huazhong Agricultural University.

### Harvest time and sample storage

*Oryza sativa* tillers were cut off under water in the early morning (between 5:30 and 6:00 am) or the previous night (between 18:30 and 19:00 pm) of the measurement day. The fresh cuts of tillers were soaked in ultra-pure water, and tillers were covered by double black plastic bags. The samples collected at night before the measurement day were conserved in ordinary ultra-pure water and sterile ultra-pure water, and half the tillers were randomly selected and recut in the morning of the measurement day to test the effect of blockage at the cut surface (Additional file 1: Fig. S3). Longer than 0.5 m *C. camphora* branches were also sampled in the early morning or previous night and conserved in ordinary ultra-pure water. It took 10–15 min to transfer the samples to the laboratory. Branches and tillers were recut under ultra-pure water in the laboratory. Their cut ends were soaked in water, and other parts were covered with double black plastic bags at least 1 h.

### Equipment settings

*K*_leaf_ was determined using evaporative flux method (EFM) reported previously [5, 22]. To minimize estimation errors, the water evaporation in the graduated cylinder without leaf under multiple conditions was quantified. The system transpiration was measured under the following four conditions: water without intervene, water surface covered by liquid wax, maintaining high humidity in the balance chamber by putting wet tissue papers, and the combination of liquid wax cover and putting wet tissue papers in the balance chamber. Based on our results (Additional file 1: Fig. S2), the combination of liquid wax cover and putting wet tissue papers in the balance chamber was adopted in the subsequent experiments due to its superior capacity to prevent water loss. In order to avoid ion deposition in leaf, ultra-pure water rather than ionic solution was adopted for leaf uptake[54, 55]. Ultra-pure water was vacuumed for 8 h to remove bubbles or directly stored overnight for *K*_leaf_ measuring of two species.

One end of low-resistance transparent tube (inner diameter = 2 mm, Oupli campany, Shanghai, China) filled with water was connected to the graduated cylinder containing with water on a balance (± 0.01 mg; Mettler MS205DU, Mettler-Toledo GmbH, Greifensee, Switzerland) (see the equipment diagram in Additional file 1: Fig. S9). Finally, water volume in cylinder was adjusted to ensure that leaves were placed 2 cm below the meniscus of the water in the cylinder for *K*_leaf_ measurement of two species.

### Changes in environmental factors

To investigate the influences of environmental factors on *K*_leaf_ estimation, three environmental factors—air temperature, ambient photosynthetically active radiation (PARa), and airflow around *O. sativa* leaf—were individually changed. The environment conditions were as follows: the temperature was set as 37 °C$\pm 1$ or 27 °C$\pm 1$; PARa at leaf surface was set as 500, 1000, or 1500 µmol m^−2^ s^−1^; the airflow around leaves was set as 1 and 0 m s^−1^.

### ***K***_leaf_ measurement

The newly- and fully-expanded leaves with 2 cm sheathes or petioles were cut from tiller under distilled water. The petioles of *C. camphora* leaves were connected to the water pipe using a hose tape. A hose tape and a cork were used to achieve seamless connection between *O. sativa* leaf sheathes and tube. Leaf was lifted higher than water surface to detect whether bubbles occurred in the connection. After leaf was placed on fish line net, leaf surface was wiped with tissue paper and irradiated by a lamp (600 W, Weichuang Company, Wuhan, China). A box fan (Comfort Zone 20 Inch Box Fan, the factory Depot Advantages, Inc, USA) was used to minimize the boundary resistance. At the same time, water weight in the graduated cylinder and the slope between weight and time were recorded every 3 s.

The water loss rate into the leaves was recorded until it was stable for a period of time (> 15 min). The detail identification of steady state was described in “Statistical analysis” section below. The temperature of the blade middle was determined as leaf temperature using a thermocouple (XimaAS877, Wanchuang electronic products Co., Ltd., Dongguan, China). Afterwards, leaf area and the final leaf water potential (ψ_final_) were measured. *K*_leaf_ was calculated according to the following formula:

$${K}_{leaf}=E/(0-{\Psi }_{\mathrm{final}})$$

All the *K*_leaf_ values were normalized to those at 25 °C considering that water viscosity varied with temperature [56]. The measurements were performed from 8:00 am to 18:00 pm since there was no correlation between measuring time and *K*_leaf_ or *E* (data not shown).

### Leaf water potentials

Upper and lower leaves adjacent to the target leaf used for hydraulic conductance measurement were cut from the tiller before *K*_leaf_ measurement, quickly put in an exhaled double-layer zip-lock bag, and placed in a foam box for water potential equilibration. Subsequently, leaf initial water potential (ψ_initial_) was detected in pressure chamber (PMS Instrument Company, Albany, OR, USA). Constant slow pressurization rate (< 0.05 MP s^−1^) was maintained during measurement. After flow measurement, the final leaf water potential (ψ_final_) was determined as described above. To investigate the influences of equilibration time on leaf water potential estimation, water potential measurements were conducted on leaves in foam box for 10, 20, 30, 60, 90, 120, 150, and 180 min. Since there was no difference in ψ_final_ under 10–60 min equilibration, a 30 min of equilibration to leaves were applied in this study.

### Liquid flow and gas flow comparison

In order to test the consistency of liquid flow rate and gas flow rate, the balance based liquid flow rate and Licor 6800 (LI-COR Inc., Lincoln, NE, USA) based gas flow rate were simultaneously measured. Leaves were quickly clamped into a 6 × 6 cm transparent chamber (Li-6800-13, LI-COR Inc., Lincoln, NE, USA) in the middle of *O. sativa* leaf or the entire leaf blade of the *C. camphora* after being put on net. In addition, a 3 × 3 cm transparent chamber (Li-6800-02) was used to clamp part *C. camphora* leaf blades. The chamber environment was set as coincident with ambient environment as possible. Auto-log was conducted with 30-s interval until a steady state was reached.

### Statistical analysis

As the flow data was typically dynamic with time, the judgment on whether the flow rate has stabilized is challenging. An effective method to restrict segment lengths of given flow data is to explicitly allow high variance of segments, and segment length restriction was achieved via the break-point penalty parameter P in ‘*dpseg*’ package. In our analysis, high P value will allow high variance of the individual segments to produce long segments. The flow rate—time curve was segmented according to P-value, and the obtained segments were marked with different color. Different segments within 1 min separated by a few outliers were deemed invalid (Fig. 4). The time of last segment was required to be longer than 15 min, and the t-test P-value of the curve slope corresponding to the last 15 min (about 300 points) was required to be larger than 0.001.

One-way analysis of variation (ANOVA) and multiple comparisons (least significant ranges, ‘*agricolae*’ package) were conducted to test the significance of different treatments. The correlation in Fig. 5 was fitted using ‘*ggpmisc*’ package. All figures were plotted using ‘*tidyverse*’ package. All of the statistics and plotting were performed in R version 3.6.1 (https://cran.r-project.org).

## Supplementary Information

**Additional file 1: Figure S1.** A literature survey on *K*_leaf_ measuring methods. **Figure S2.** The water loss from the cylinder without leaf under different preventions. **Figure S3.** Influences of sample storage time and recutting on initial leaf water potential. **Figure S4.** Dynamic water flow rate measured by a Licor 6800 and a balance in the same leaf. **Figure S5.** Oscillation of water flow rate, transpiration rate, and stomatal conductance. **Figure S6.** Temperature effects on water flow rate estimation. **Figure S7.** Modeled final leaf water potential and *K*_leaf_ changes over the time. **Figure S8.** Effects of equilibration time on leaf water potential estimation. **Figure S9.** Experimental setup used in determining *K*_leaf_. **Figure S10.** Meteorological data of rice growing season.

**Additional file 2: Table S1.** The meta-data sheet of reported *K*_leaf_.

**Additional file 3: Table S2.** All tidy data used in the main figures.

**Additional file 4: Table S3.** All raw flow data used in the in the main figures.

## Data Availability

The datasets supporting the findings of this study are available within the paper and within its additional files published online.
