# Supplementary material for: Evaporative flux method of leaf hydraulic conductance estimation: sources of uncertainty and reporting format recommendation
Source: Plant Methods. 2022 May 12;18:63. doi: 10.1186/s13007-022-00888-w (PMC9097237; doi:10.1186/s13007-022-00888-w)
Supplement: Supplementary file 1 — Additional file 1: Figure S1. A literature survey on Kleaf measuring methods. Figure S2. The water loss from the cylinder without leaf under different preventions. Figure S3. Influences of sample storage time and recutting on initial leaf water potential. Figure S4. Dynamic water flow rate measured by a Licor 6800 and a balance in the same leaf. Figure S5. Oscillation of water flow rate, transpiration rate, and stomatal conductance. Figure S6. Temperature effects on water flow rate estimation. Figure S7. Modeled final leaf water potential and Kleaf changes over the time. Figure S8. Effects of equilibration time on leaf water potential estimation. Figure S9. Experimental setup used in determining Kleaf. Figure S10. Meteorological data of rice growing season. [file 13007_2022_888_MOESM1_ESM.pdf]

Evaporative flux method of leaf hydraulic conductance estimation: sources of uncertainty and reporting format recommendation

Running title: Evaluating evaporative flux method of K_{leaf} estimation

Xiaoxiao Wang, Jinfang Zhao, Jianliang Huang, Shaobing Peng, Dongliang Xiong*

*Corresponding author, Dr. Dongliang Xiong, Tel: +86 (0)27 87288961, Email: dlxiong@mail.hzau.edu.cn

National Key Laboratory of Crop Genetic Improvement, MOA Key Laboratory of Crop Ecophysiology and Farming System in the Middle Reaches of the Yangtze River, College of Plant Science and Technology, Huazhong Agricultural University, Wuhan, Hubei 430070, China

Supplementary

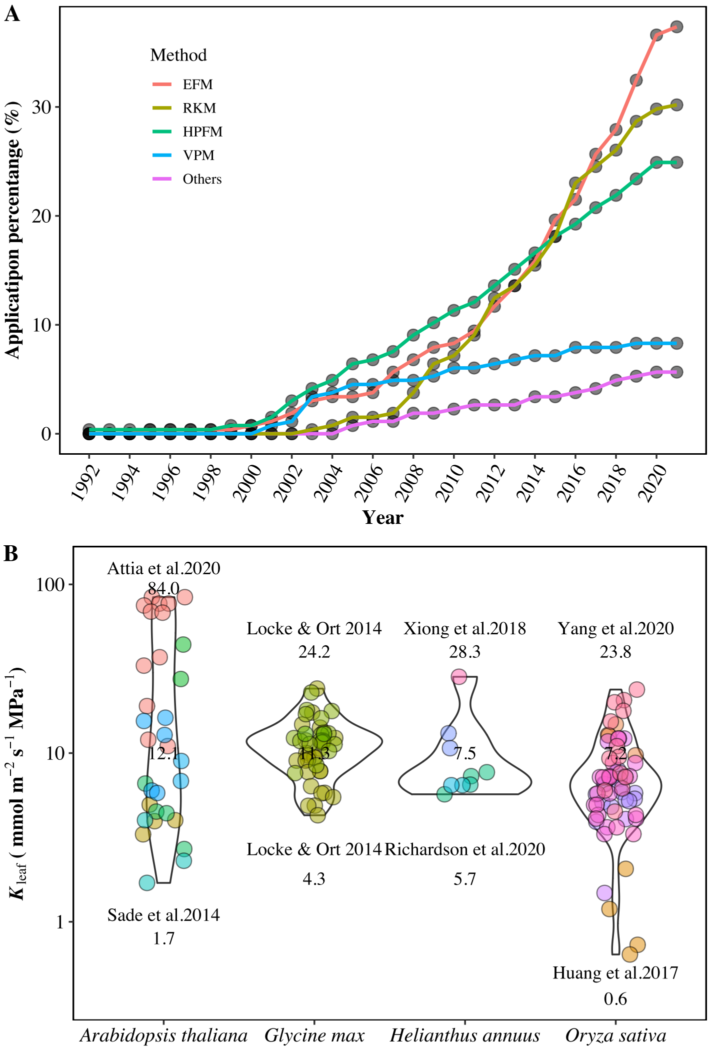

Fig S1. (A) Leaf hydraulic conductance (K_{leaf}) estimation methods used in literature. (B) Variation of K_{leaf} values of the four top estimated species. In panel (A), EFM, RKM, HPFM and VPM represent evaporation flux method, rehydration kinetics methods, the high-pressure flowmeter, and vacuum pump method, respectively. In panel (B), the different colors of points represent different studies and the studies reported the maximum and minimum K_{leaf} values were marked.

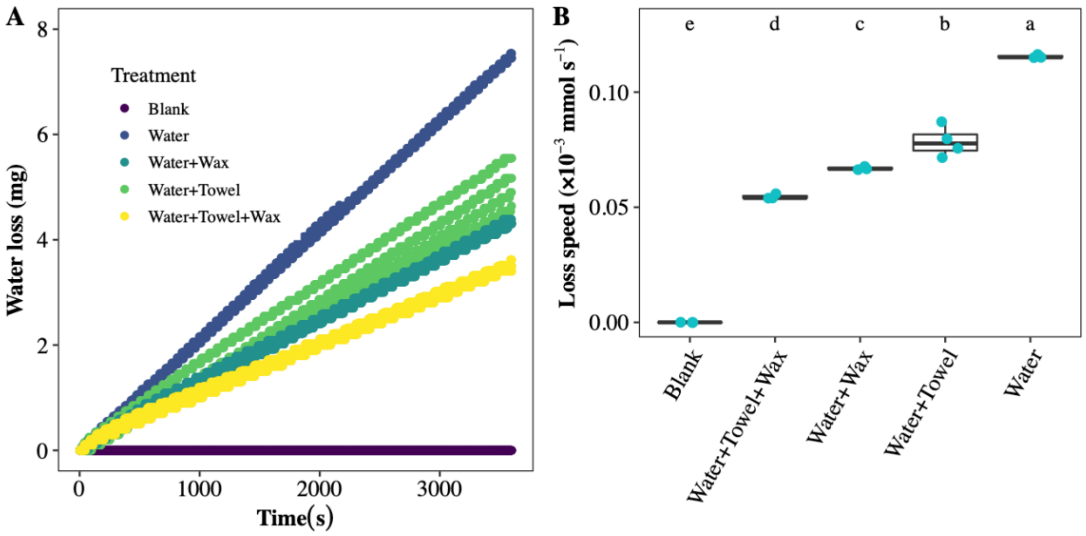

Fig S2. (A)The water loss from the cylinder with or without wax cover and wet towel in the balance chamber. (B) Slopes of water loss to measurement time under different extra water loss preventions: Blank, no water on balance; Water, only water on balance; Water+Towel, wet towel put in balance chamber; Water+Wax, liquid wax covered on water surface; Water+Towel+Wax, wet towel put in balance chamber and liquid wax covered on water source surface. Different letters represent statistical significance ($P < 0.05$).

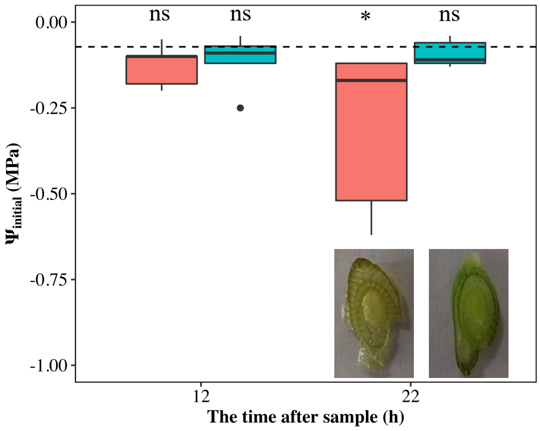

Fig S3. Influences of sample storage time and recutting on initial water potentials ($\psi_{initial}$) of *O. sativa* leaves sampled at previous night. All samples were cut under water and rehydrated after collection. Different rehydration time and whether recut were applied. Dashed line represents the mean $\psi_{initial}$ value (-0.072 MPa) after rehydration 1 h. Red boxplot represents $\psi_{initial}$ of rehydrated samples without recutting. Blue boxplot indicates the $\psi_{initial}$ of rehydrated samples recut on the measurement day morning. Bottom illustrations show the cut surface at corresponding time. (ns: no difference between box and $\psi_{initial}$ value after rehydration 1 h; *, $P < 0.05$)

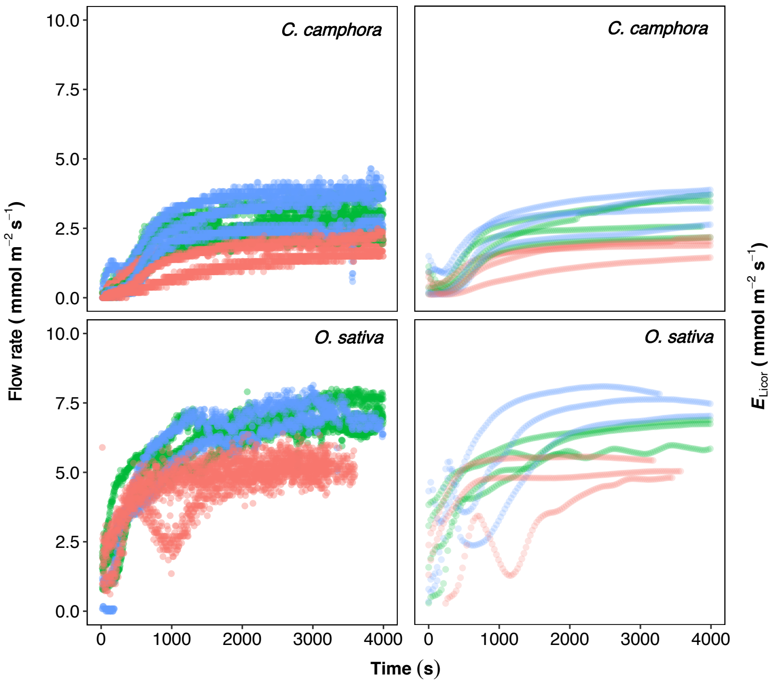

Fig S4. Dynamic flow rate of *O. sativa* and *C. camphora* measured by balance or by Licor6800 (E_{licor}) (raw data unfiltered) under multiple photosynthetically active radiation (PARa). Red point, PARa = 500 μmol m^{-2} s^{-1}; Green point, PARa = 1000 μmol m^{-2} s^{-1}; Blue point, PARa = 1500 μmol m^{-2} s^{-1}

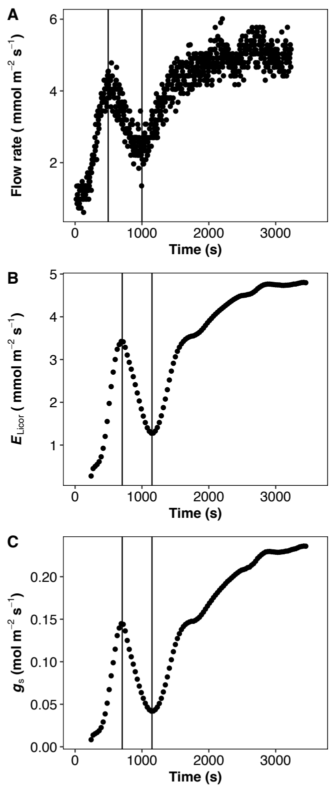

Fig S5. (A) Oscillation of liquid phase water flow rate, (B) gas phase transpiration rate (E_{Liocr}) and (C) stomatal conductance (g_s) after *O. sativa* leaf lamped in a Liocr chamber. The vertical solid line presents the time at crest and trough in oscillation.

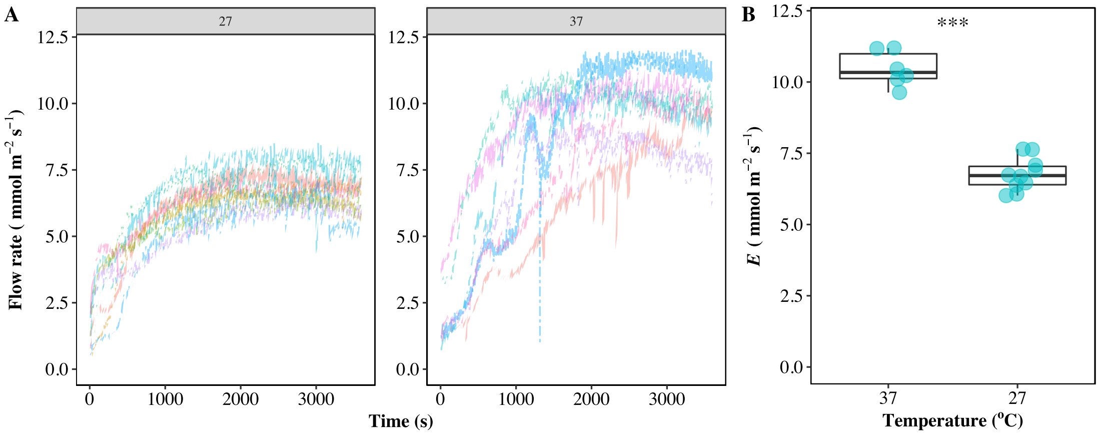

Fig S6. (A) Dynamic flow rate over time and (B) steady state flow rate (*E*) of *O. sativa* leaves measured in two air temperature conditions (27 ºC and 37 ºC, raw data unfiltered). (***, $P < 0.001$)

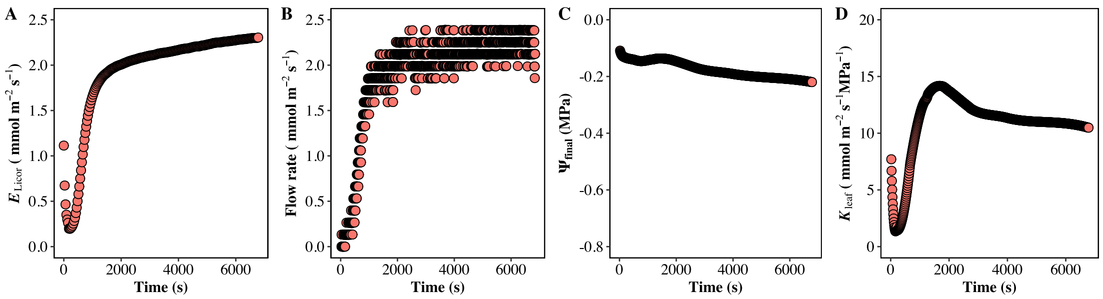

Fig S7. Modeled leaf water potential (ψ_{final}) and leaf hydraulic conductance (K_{leaf}) by dynamically fitting the water loss from the leaf by transpiring (E_{Licor}) and the flow rate enter the *C. camphora* leaf. (A) Gas phase water flow dynamics. (B) Liquid water flow dynamics. (C) Modeled dynamic ψ_{final} (D) Modeled leaf hydraulic conductance dynamics. Simulation method followed to Martins et al., 2016. Dynamic ψ_{final} was calculated as: $\psi_{final,t-10s} = \psi_{leaf,t} - \frac{(E_{Licor,t-10s} - Flow\ rate_{,t-10s})*10}{C_{leaf}}$. K_{leaf} values were inferred from E_{Liocr} values and reconstructed instantaneous ψ_{final} values as $K_{leaf} = \frac{E_{Liocr}}{\psi_{final}}$.

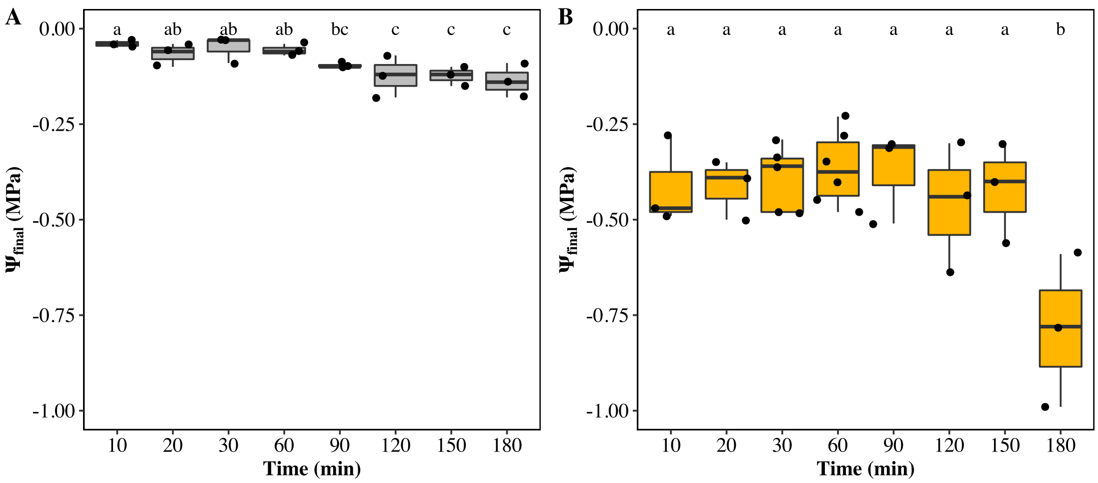

Fig S8. Final water potential of *O. sativa* leaves (ψ_{final}) with multiple equilibration time in the double plastic zip lock bags. (A) *O. sativa* leaves acclimated to dark (grey box) and (B) 1000 μmol m^{-2} s^{-1} PARa (yellow box) in advance. Different lower-case letters indicate significant differences at 0.05 level.

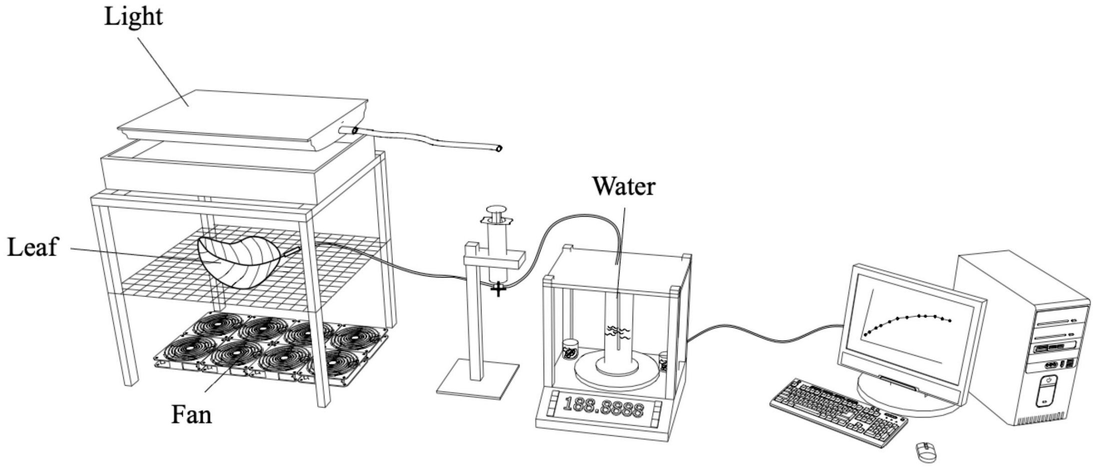

Fig S9. Experimental setup used in determining the leaf hydraulic conductance (K_{leaf}). Leaves were laid on the transparent net with airflow by fan and radiation by lamp. The water uptake was measured by balance which connecting to a computer. A syringed was used to adjust height gradients between the leaf blade and the water surface in the cylinder. The relative wet micro-environment in balance chamber was created by putting wet tissues.

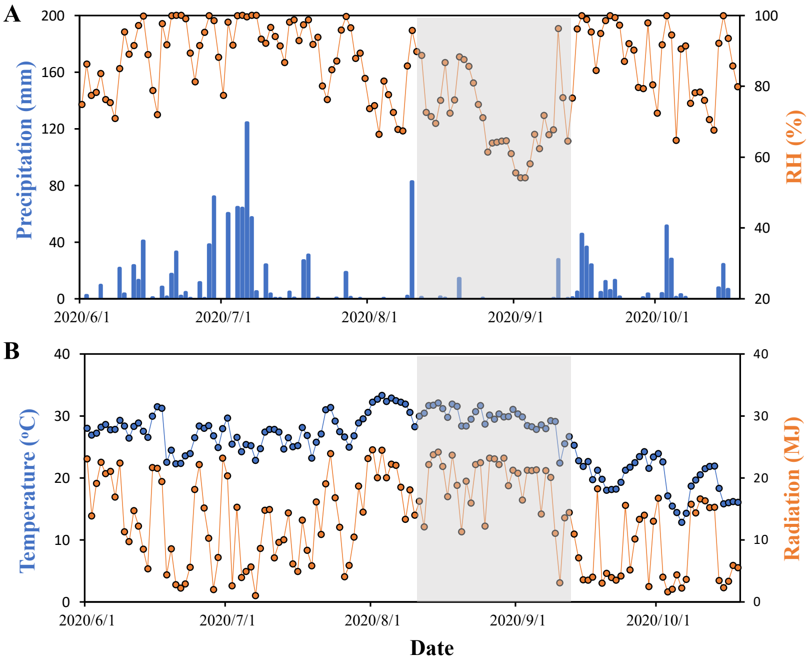

Fig S10. Daily precipitation, relative humidity (A), and the average air temperature and radiation (B) during rice growing season. The gray area represents the sampling date.

1. Martins, S. C., McAdam, S. A., Deans, R. M., DaMatta, F. M. and Brodribb, T. J. Stomatal dynamics are limited by leaf hydraulics in ferns and conifers: results from simultaneous measurements of liquid and vapour fluxes in leaves. Plant, Cell and Environment. 2016; 39: 694-705.
